# Improved Cycle Stability of LiSn Alloy Anode for Different Electrolyte Systems in Lithium Battery

**DOI:** 10.3390/nano11020300

**Published:** 2021-01-25

**Authors:** Jin Lou, Kanghua Chen, Nachuan Yang, Yi Shuai, Changjun Zhu

**Affiliations:** 1 Light Alloy Research Institute, Central South University, Changsha 410083, China; jinlou@csu.edu.cn (J.L.); kanghuachen@csu.edu.cn (K.C.); shuaiyi@hnie.edu.cn (Y.S.); 2 Collaborative Innovation Center of Advance Nonferrous Structural Materials and Manufacturing, Central South University, Changsha 410083, China; cyflz@mail.ustc.edu.cn; 3 State Key Laboratory of High Performance Complex Manufacturing, Central South University, Changsha 410083, China; 4 Department of Mechanical Engineering, Hunan Institute of Engineering, Xiangtan 411104, China

**Keywords:** lithium battery, alloy anode, dendrite suppress, solid state electrolyte

## Abstract

Lithium metal anode still confronts a series of problems at the way to commercialization though it has advantages in high energy density. The formation of Li dendrite is the major limitation need to be conquered. Here, a facile and simple LiSn alloy anode prepared by a direct metallurgy method is fabricated and evaluated in both liquid electrolyte and solid electrolyte. Structural analysis and electrochemical measurements reveal the promoted ionic transference of interface and enhanced cycling stability in different electrolyte systems, without dendrite formation. Furthermore, the application of this simple and sustainable LiSn alloy can be extended to more alloy anode and might unlock the next-generation anode in the future.

## 1. Introduction

The development of lithium-ion batteries (LIBs) continued to draw worldwide attention since it was first commercialized in portable consumer electronics by SONY in 1991 [1]. The ever increasing energy demand of high-energy storage expanded the stage of high-capacity anode materials. Li-metal anode, possessing high theoretical specific capacity of 3860 mAh g^−1^ and lowest-redox potential of −3.04 V (vs. standard hydrogen electrode) has gradually been approved to be the best candidate in the next generation of LIBs [2,3,4]. However, there are still issues and challenges including the poor cycle performance and the volume expansion during cycling [5,6,7]. The growth of Li dendrite will eventually cause fire by piercing the separator which is the principal bottleneck for the commercialization of such batteries. In addition, the formation of dendrite produces ‘dead Li’ by destroying the solid electrolyte interface (SEI) which will results in the decrease of Coulombic efficiency and deterioration of interface, especially in solid state electrolyte [8,9,10]. Various strategies were adopted to suppress or eliminate the dendrites [11,12]: mixing of ionic additives to solvent [13,14,15], coating of hybrid layer [16,17] on Li surface, or 3D micro-structuration of Li metal [18]. Although these methods show advantages in cycle performance, their practicability are still doubtful because of low economical efficiency.

Recently, a large number of studies shows that LiM (M = Si, Sn, Zn, In, etc.) alloy can accelerate the diffusion and transference of Li-ion at the interface by Kirkendall effect [19,20,21]. The distinctly decreased impedance of alloyed anode is attributed to suppressing dendrite and conductive alloy frame structure in SEI. However, many alloying processes are carried out by an in-situ reaction or ionic additives to solvent [22]. The alloying layer fabricated by interfacial alloying can efficiently promote the cycle stability [23]; however, the process of alloying is difficult to control. Besides that, the electrolyte additives can hardly be commercialized, because the content of electrolyte additives is unstable during the charge–discharge process. The LiSn anodes were recently examined using a conductive polypyrrole (PPy) on the Sn nanoparticle [24]. The composites in LixSn@PPy|LiFePO_4_ cells were stable for over 80 cycles. In this work, direct metallurgy method was adopted in preparation of LiSn alloy. To recognize the characteristics of metallic electrode interface and mechanism of dendrite formation of Li, XRD and SEM tests were carried out. Furthermore, the interfacial stability in sulfide solid state electrolyte was also investigated to unveil wider application of alloying anode.

## 2. Experimental Procedure

### 2.1. Preparation of Li_0.98_Sn_0.02_ Alloy Anode

The Li_0.98_Sn_0.02_ alloy was synthesized by direct metallurgy method. In an Ar-filled glove box, an entirely melted Li metal (99.9%) was added into a prepared tin metal (99.9%) in a molar ratio of 49:1. In the process of the experiment, we found that excessive Sn will destroy the stability of the electrode interface because of large volume expansion of intermetallic compound. The ratio of Li and Sn was obtained by the adjustment during the metallurgy. While at the ratio of 49:1, it could be seen that two ingredients melted gradually and formed the homogeneous melt. Thus, the molar ratio of 49:1 was selected. Sufficiently stirring was required to obtain the homogeneity of the structure. After cooling to room temperature, the alloy ingots were rolled and punched into pieces as prepared.

### 2.2. Preparation of Sulfide Solid State Electrolyte

Sulfide solid state electrolyte was synthesized using a planetary ball mill method. Li (99.9%), S (99%), P_2_S_5_ (99.9%), and LiCl (99.9%) were weighed and mixed in a molar ratio of Li:S:P_2_S_5_:LiCl to 10:6:1:2 in an Ar-filled glove box, placed into a stainless steel tank with ZrO_2_ balls and mixed for 20 h. The mixed powder (LiSPCl) was then sealed in a stainless steel tank and heat-treated at 500 °C for 12 h, and cooled naturally to room temperature. LiSPCl/PEO/LiClO_4_ (18:1:1 in a weight ratio) was prepared via liquid-phase process. As-prepared LiSPCl was milled at 300 rpm for 5 h to obtain a finer and uniform particle in advance. The mixture of PEO (Mw = 600,000, Sigma-Aldrich, Shanghai, China) and LiClO_4_ was dissolved in anhydrous acetonitrile (Aladdin, Shanghai, China) and stirred for 24 h in order to obtain the homogeneous solution. Subsequently, the milled LiSPCl was added into the above mixed solution and stirred for 6 h and the slurry was dried at 60 °C for 24 h and the LiSPCl/PEO/LiClO_4_ composite solid electrolyte was finally prepared.

### 2.3. Morphology and Electrochemical Characterization

The electrochemical performance of the as-prepared electrode was characterized using CR 2032 coin-type half-cells, polypropylene Celgard 2500 as the separator (Celgard), 1 M LiPF_6_ in ethylene carbonate and diethyl carbonate (EC:DEC = 1:1) as the liquid electrolyte (DoDoChem Co. Ltd., Suzhou, China). Galvanostatic charge–discharge measurements were performed by Wuhan Land system at room temperature. The electrochemical tests were performed at current densities from 0.4 mA cm^−2^ to 2 mA cm^−2^. X-ray diffraction (XRD) patterns were recorded on a Bruker D8 Advance (Cu-Kα) diffractometer (Bruker, Germany) operated at 40 kV and 200 mA. Samples were protected by polyimide film to avoid contamination of water and oxygen in the air during measurement. Scanning electron micrographs (SEM) and energy dispersive spectral (EDS) measurements were performed via a FEI Nova Nano SEM 230 scanning electron microscope (FEI, Hillsboro, OR, USA). AC impedance spectra were obtained on Agilent 4294A (Agilent, Shanghai, China) at frequencies from 40 Hz to 110 MHz.

## 3. Results and Discussion

**Figure 1:**
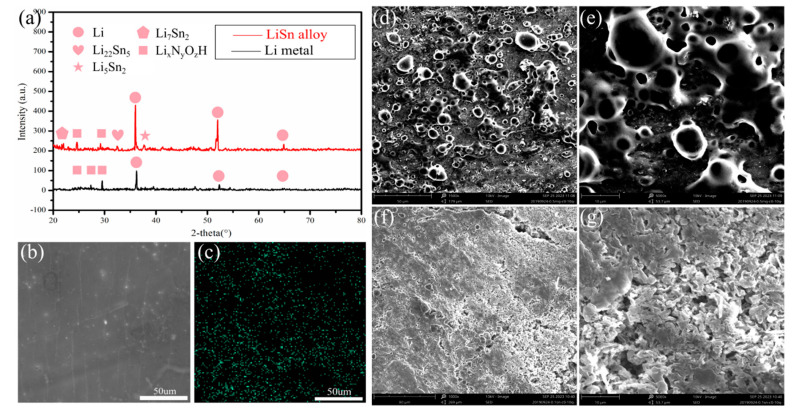
X-ray diffraction patterns (**a**), EDS analysis (**b**,**c**), SEM images of pristine Li anode in 20th cycle (**d**,**e**) and SEM images of LiSn anode in 20th cycle (**f**,**g**).

**Figure 2:**
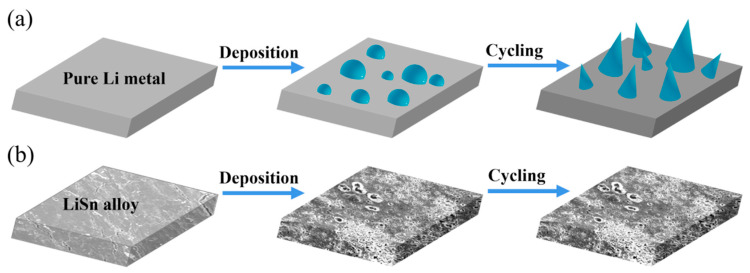
Schematic sketches of the deposition and cycling process on the (**a**) pure Li metal and (**b**) LiSn alloy anode.

The structural characterization of LiSn alloy is shown in Figure 1. The results show that the alloy anode was successfully prepared. The XRD patterns of both LiSn alloy and pure Li electrode were measured and shown in Figure 1a. The pure Li has three peaks situated at 2θ = 36.2°, 52°, and 65° (JCPDS 015-0401), which correspond to the crystal planes of Li. After electrodeposition, several peaks at 2θ = 32.7°, 38°, 21.7°, 22.3°, and 22.7° were detected in LiSn electrodes. The peaks coincide well with the intermetallic alloy phase Li_22_Sn_5_ (JCPDS 015-0401), Li_5_Sn_2_ (JCPDS 029-0839), and Li_7_Sn_2_ (JCPDS 029-0837), indicating that the LiSn alloy films mainly consists of three phases including Li_22_Sn_5_, Li_5_Sn_2_, and Li_7_Sn [2]. Li_x_N_y_O_z_H was also detected because of the inevitable oxidation of polyimide film. The EDS mapping analysis of LiSn alloy certified the uniform distribution of Sn element (Figure 1b,c). Figure 1d,e shows the microscopic morphology of pristine Li anode surface in 20th cycle. Evident clusters found on the coarse surface proved that the structure of pure Li anode is unstable during the process of dissolution and deposition. Figure 1f,g shows the deposition state of LiSn alloy electrode in 20th cycle. A flat and smooth structure was detected on the surface with uniform deposition on the image. It is known that there is always an uneven structure on the surface of pure Li metal. The charge on Li always concentrates on the convex structure where the Li dendrites form (Figure 2a). Thus, in alloyed electrode, the ionic state Li will prevent the formation of Li dendrite by inducing the deposition of Li metal. As can be seen in Figure 2b, in LiSn alloy, the presence of a LiSn compound can promote uniform deposition and inhibit the growth of Li dendrites (Figure 2b).

**Figure 3:**
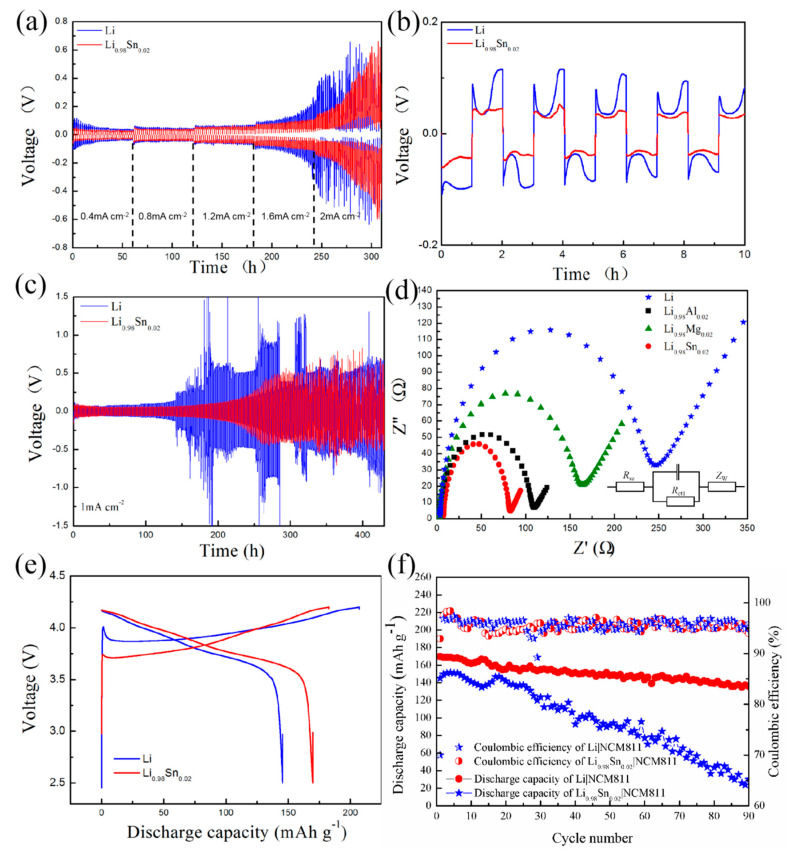
(**a**) Rate performance of liquid electrolyte system of Li|Li and LiSn|LiSn at the current densities ranging from 0.4 to 2 mA cm^−2^. (**b**) The enlarged typical profiles ranged from 0 to 10 h. (**c**) Cycle performance of Li|Li and LiSn|LiSn at a current density of 1 mA cm^−2^. (**d**) Nyquist plots for Li, LiSn, and other LiM (M = Al, Mg) alloys, respectively. (**e**,**f**) The cycle performance of full cell using commercial cathode material (NCM811).

In order to unveil the cycling stability of LiSn alloy anode, galvanostatic symmetric cells of Li|Li and LiSn|LiSn were assembled and tested at the current density from 0.4 to 2 mA cm^−2^ with a fixed plating/stripping time of 1 h. The symmetric cell of Li|Li showed high overpotential during the first several cycles because of the strong inertness of the passivation layer on Li surface. By contrast, LiSn|LiSn cell possess stable interface and the overpotential goes steady. LiSn|LiSn cell could stably cycle for 230 h with an overpotential of about 40 mV at 1 mA cm^−2^ while Li|Li symmetric cell merely delivered limited circuit after 140 h with an overpotential of about 100 mV, as shown in Figure 3c. At different current densities ranging from 0.4 to 2 mA cm^−2^, as shown in Figure 3a, LiSn|LiSn symmetric cell presented better cycle performance with lower stable overpotential than Li|Li cell. It can be learned that LiSn anode is more favorable for inducing uniform Li metal deposition and growth. At 1 mA cm^−2^, the enlarged typical profiles ranged from 0 to 10 h, as shown in Figure 3b. To further examine the electronic properties of the LiSn alloy, we performed the electrochemical impedance spectroscopy (EIS) measurements of LiSn, LiAl, LiMg, and Li electrodes after the first lithiation, as depicted in Figure 3d. The semicircles and straight lines in the Nyquist plots represent charge transfer resistance (R_ct_) and diffusion of Li-ion, respectively. It is estimated that the charge transfer resistance of LiSn, LiAl, LiMg, and Li are 80 Ω, 110 Ω, 160 Ω, and 250 Ω, respectively. It can be inferred that, compared with pure Li electrode, alloyed anode can enhance electronic conductivity. On the other hand, LiSn alloy possess the optimum conductivity among the testing LiM (M = Sn, Al, and Mg) alloys. This could be ascribed to the fast electron diffusion LiSn layer on the interface, which effectively promotes electronic conductivity and reaction kinetics. According to the results shown in Figure 3e,f, it can be concluded that, as a commercial cathode material (NCM811) is used, LiSn alloy anode also exhibits better cycle performance in full cell.

**Figure 4:**
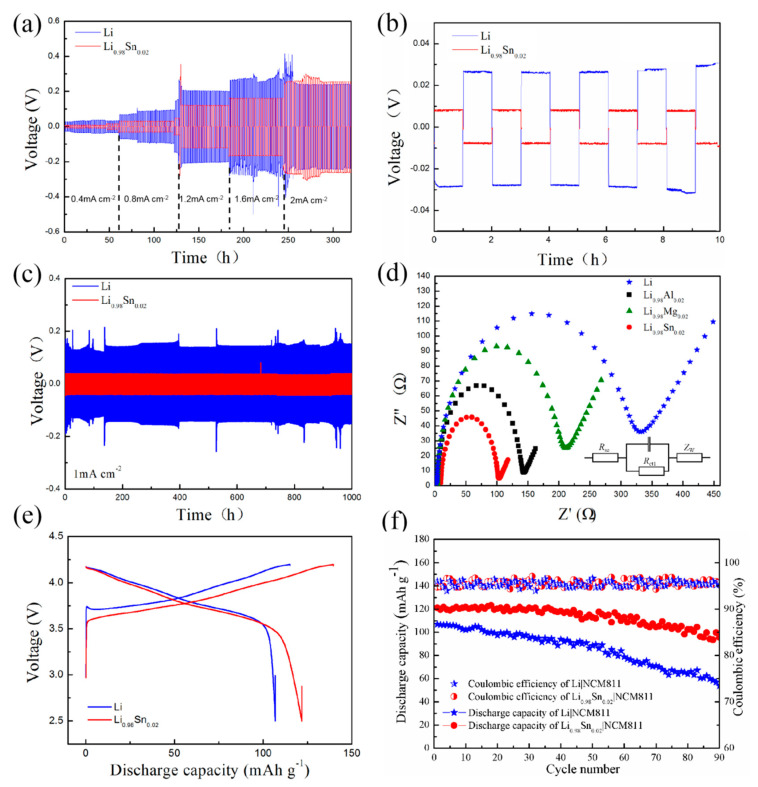
(**a**) Rate performance of solid electrolyte of Li|Li and LiSn|LiSn at the current densities ranging from 0.4 to 2 mA cm^−2^. (**b**) The enlarged typical profiles ranged from 0 to 10 h. (**c**) Cycle performance of Li|Li and LiSn|LiSn at a current density of 1 mA cm^−2^. (**d**) Nyquist plots for Li, LiSn, and other LiM (M = Al, Mg) alloys, respectively. (**e**,**f**) The cycle performance of full cell using commercial cathode material (NCM811).

In order to prove the positive effect of alloy anode, similar electrochemical tests were carried out in solid state electrolyte. According to the results shown in Figure 4, it can be seen that in sulfide solid state electrolyte system, LiSn alloy anode presented superior stability than pure Li anode. At different current densities (Figure 4a), LiSn|LiSn cell could stably cycle with lower overpotential than Li|Li cell. At 1 mA cm^−2^, the overpotential of LiSn|LiSn cell was only about 15 mV after 1000 h. The enlarged typical profiles shown in Figure 4b indicated that the overpotential of LiSn alloy was much lower than that of pure Li metal. For the corresponding symmetric cell with Li electrode, its overpotential increased rapidly after only 130 h from about 50 mV to 100 mV (Figure 4c). The electrochemical impedance spectroscopy (EIS) measurements of LiSn, LiAl, LiMg, and Li electrodes were carried out after the first lithiation, depicted in Figure 4d. It is estimated that in solid state electrolyte system, LiSn alloy also possess the optimum conductivity among the testing LiM (M = Sn, Al, and Mg) alloys. It is deduced that LiSn alloy can suppress the corrosion of Li in sulfide solid electrolyte and exhibited enhanced cycling stability in sulfide solid electrolyte.

Consequently, a full cell of LiSn|NCM811 was evaluated in both the liquid and solid state electrolyte systems, and separately shown in Figure 3e,f and Figure 4e,f. It can be seen that owing to promoted ionic transference of interface, the cycling stability was enhanced evidently. At 0.1C, the full cell had a capacity of 170 mA h g^−1^ in liquid electrolyte and 122 mA h g^−1^ in solid electrolyte. After 90 cycles, the capacity retention was approximately 82% in both electrolyte systems.

## 4. Conclusions

In summary, we have demonstrated a LiSn alloy as a high performance anode fabricated by a direct metallurgy method. The XRD analysis of the LiSn alloy electrode confirmed the formation of Li_22_Sn_5_, Li_7_Sn_2_, and Li_5_Sn_2_ phases in Li metal. Besides that, the SEM analysis of cycled LiSn electrode demonstrated the positive effect in deposition, resulting in a smooth surface with no dendrite. With these advantages, LiSn alloy anode exhibits superior stability in both liquid electrolyte and solid state electrolyte. We believe that this facile and simple design could be enlightened for more novel and practical alloy anodes in different electrolyte systems.
